# Prediction of Wilms’ Tumor Susceptibility to Preoperative Chemotherapy Using a Novel Computer-Aided Prediction System

**DOI:** 10.3390/diagnostics13030486

**Published:** 2023-01-29

**Authors:** Israa Sharaby, Ahmed Alksas, Ahmed Nashat, Hossam Magdy Balaha, Mohamed Shehata, Mallorie Gayhart, Ali Mahmoud, Mohammed Ghazal, Ashraf Khalil, Rasha T. Abouelkheir, Ahmed Elmahdy, Ahmed Abdelhalim, Ahmed Mosbah, Ayman El-Baz

**Affiliations:** 1 Bioengineering Department, University of Louisville, Louisville, KY 40292, USA; 2 Urology and Nephrology Center, Mansoura University, Mansoura 35516, Egypt; 3 Department of Biology, Berea College, Berea, KY 40292, USA; 4 Electrical, Computer, and Biomedical Engineering Department, Abu Dhabi University, Abu Dhabi 59911, United Arab Emirates; 5 College of Technological Innovation, Zayed University, Abu Dhabi 144534, United Arab Emirates; 6 Department of Urology, West Virginia University, Morgantown, WV 26506, USA

**Keywords:** features engineering, machine learning, preoperative chemotherapy, Wilms’ tumor

## Abstract

Wilms’ tumor, the most prevalent renal tumor in children, is known for its aggressive prognosis and recurrence. Treatment of Wilms’ tumor is multimodal, including surgery, chemotherapy, and occasionally, radiation therapy. Preoperative chemotherapy is used routinely in European studies and in select indications in North American trials. The objective of this study was to build a novel computer-aided prediction system for preoperative chemotherapy response in Wilms’ tumors. A total of 63 patients (age range: 6 months–14 years) were included in this study, after receiving their guardians’ informed consent. We incorporated contrast-enhanced computed tomography imaging to extract the texture, shape, and functionality-based features from Wilms’ tumors before chemotherapy. The proposed system consists of six steps: (i) delineate the tumors’ images across the three contrast phases; (ii) characterize the texture of the tumors using first- and second-order textural features; (iii) extract the shape features by applying a parametric spherical harmonics model, sphericity, and elongation; (iv) capture the intensity changes across the contrast phases to describe the tumors’ functionality; (v) apply features fusion based on the extracted features; and (vi) determine the final prediction as responsive or non-responsive via a tuned support vector machine classifier. The system achieved an overall accuracy of 95.24%, with 95.65% sensitivity and 94.12% specificity. Using the support vector machine along with the integrated features led to superior results compared with other classification models. This study integrates novel imaging markers with a machine learning classification model to make early predictions about how a Wilms’ tumor will respond to preoperative chemotherapy. This can lead to personalized management plans for Wilms’ tumors.

## 1. Introduction

Wilms’ tumor (WT) is the most common primary renal tumor in children, and it is the second most prevalent intra-abdominal malignancy in children. It accounts for over 90% of all kidney tumors in children under the age of 18 years and represents 4% of all childhood malignancies. The annual incidence of WT in the United States is approximately 500 to 600 children under the age of 15, with a peak incidence between 3 and 4 years of age [1,2].

The initial imaging investigation of a child presenting with an abdominal mass is usually via an abdominal ultrasound; however, axial imaging with magnetic resonance imaging (MRI) or computerized tomography (CT) is key to obtain more detailed anatomic information that is crucial for diagnosis and treatment planning. For instance, a CT can give an idea about the extent of the local tumor, the presence of a tumor thrombus, and possible lymph node involvement. The initial enthusiasm for using CT scans to accurately stage childhood renal tumors preoperatively waned with subsequent studies showing inaccuracies in the radiologic staging of WT. The accurate staging of WT is based on data gained during surgical exploration and histopathologic examinations of nephrectomy specimens [3,4,5].

Modern care of WT relies heavily on multimodal therapy that combines surgery, chemotherapy, and occasionally, radiation therapy. This modern multimodal approach has been refined by the efforts of the Children’s Oncology Group (COG) and the Société internationale d’oncologie pédiatrique- (SIOP). The COG advocates upfront surgical excision, whereas the SIOP recommends systematic preoperative chemotherapy protocol, even in the absence of locally-advanced or metastatic disease. Preoperative chemotherapy results in reduction of tumor size, facilitates surgical resection, and lower the chance of tumor spillage during surgery. Nevertheless, preoperative chemotherapy is not without its disadvantages. In addition to the morbidity of chemotherapeutic agents, preoperative chemotherapy can lead to the loss of important staging information, such as eradicating neoplastic cells from lymph nodes with subsequent tumor downstaging. Systematic treatment of solid childhood renal tumors with preoperative chemotherapy without histopathological confirmation can expose children with benign renal tumors to undergo unnecessary chemotherapy and exposes those with a different malignant disease to a potentially inappropriate chemotherapy protocol. Further, tumor progression or rupture may occur while receiving chemotherapy and waiting for surgery [6]. Taskinen et al. [7] studied the effect of preoperative chemotherapy in 52 WT patients. The median tumor volume reduction after preoperative chemotherapy was 68%, but in three cases, tumor volume increased by more than 10% during treatment. Similarly, Ora et al. [8] examined WT response to preoperative chemotherapy and concluded that out of the 1090 patients studied, only 41% responded, whereas 53% had stable disease and 5% had tumor progression after preoperative chemotherapy. Therefore, there is a critical unmet demand for a non-invasive modality that can accurately predict tumor responsiveness to preoperative chemotherapy. If such a modality exists, patients who are less likely to benefit from preoperative chemotherapy can be offered upfront surgery and avoid the unnecessary morbidity of preoperative chemotherapy.

Recently, there has been growing interest in the use of AI, machine learning (ML), and the rapid advances in medical imaging to support clinical decision-making in patients with Wilms’ tumor, as well as other renal tumors. These techniques rely on the extraction of quantitative information from different imaging sources and combining this information in mathematical algorithms to provide diagnostic or prognostic information [9,10]. For instance, Xiao-Hui Ma et al. [11] developed a ML model based on contrast-enhanced CTs to preoperatively predict Wilms’ tumor stage in 118 patients. In that study, 1781 imaging-based features were extracted from the region-of-interest (ROI) of each tumor. Their dataset was split into training and testing sets with an 8:2 ratio. Using the support vector machine model (SVM), a total of 48 patients were identified as “stage I” and 70 were labeled as “non-stage I”, with an accuracy of 79%. Misch et al. [12] used pre-therapy FDG-PET to evaluate WT tumor response to preoperative chemotherapy. The response assessment was performed by MRI examination of the primary tumor site to assess tumor size reduction. In a similar perspective, Zheng et al. [13] performed imaging analysis to validate a CT-based monogram to preoperatively predict clear cell renal cell carcinoma grades. CT images were taken for 258 patients, and radiomics features were extracted from the arterial phase. Using the LASSO regression model of a training set of 143 tumors and a validation set of 115 tumors, the ROC and calibration curves were illustrated to determine the performance of the radiomics nomogram in both sets. A radiomic signature consisting of 20 features showed promising performance in differentiating between nuclear grades in the training (AUC = 0.929) and validation (AUC = 0.876) sets. Kim et al. [14] explored the power of ML in predicting the late recurrence of renal cell carcinoma that occurs 5 years after surgery. Conducting the study on eight different ML algorithms to classify 2965 patients as “late recurrence” or “non-recurrence”, the AdaBoost model reported the top performance with an accuracy of 79.9%, and an F-1 score of 0.609. Seven markers were selected, namely, tumor size, histological type, operation type, operative methods, pathological tumor stage, pathological node stage, and lymphovascular invasion, to show a significant difference (*p* < 0.05) between late recurrence and non-recurrence groups.

As far as we know, AI-based systems to predict Wilms’ tumor responses to preoperative chemotherapy have not yet been proposed. In addition, there is no reported system to consider the textural, functional, and shape features to assess Wilms’ tumor characteristics. To overcome these limitations, we are proposing a new computer-aided prediction (CAP) system that consists of six steps to predict Wilms’ tumor response to preoperative chemotherapy, based on contrast-enhanced CT images with three phases: (1) pre-contrast; (2) portal-venous; and (3) delayed-excretory phase. The proposed system extracts both first- and second-order textural features, as well as shape and functional features.

## 2. Materials and Methods

**Figure 1:**
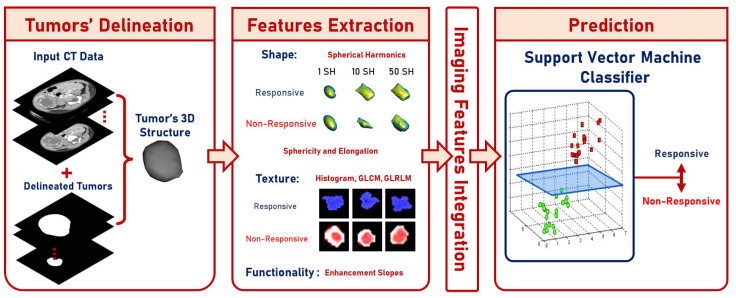
Graphical presentation of the suggested framework.

This paper shows a designed CAP system (presented in Figure 1) used to classify the effect of preoperative chemotherapy on the histologic features of Wilms’ tumor. The proposed framework follows six steps to predict the chemotherapy response: (a) delineate the tumor images throughout the three contrast phases; (b) extract shape features through elongation, sphericity, and a new parametric spherical harmonics (SHs) model using the segmented Wilms’ tumor images; (c) estimate first- and second-order textural features; (d) calculate the functionality-based features; (e) apply feature integration on the extracted groups of features; and (f) predict the final decision and calculate the corresponding performance metrics based on the optimal classifier. The final prediction can be either responsive (regression ≥30%) or non-responsive (progression or regression <30%). The main contribution of this study is that it integrates different characteristics of contrast-enhanced CT images of Wilms’ tumors before preoperative chemotherapy and can predict whether a patient will respond to the therapy.

### 2.1. Data

**Figure 2:**
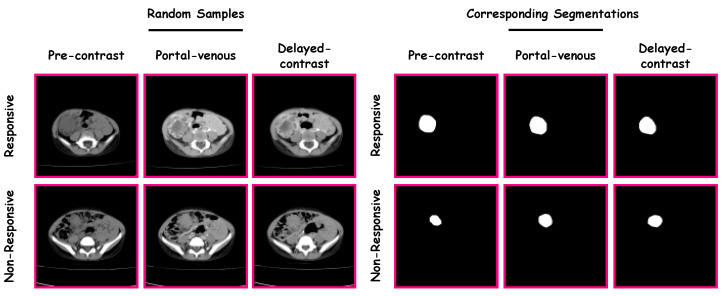
Samples from the dataset and their corresponding segmentations.

The proposed CAP system was verified and validated using data from the Urology and Nephrology Center of Mansoura University, Egypt. The study included 63 patients, where 17 were “non-responsive” and 46 were “responsive”. Patients ranged from 6 months to 14 years of age (4.31±2.82 years). The guardian of each patient gave their informed consent for participation in this study. Each patient had undergone contrast-enhanced CT with three phases (i.e., a pre-contrast phase, a portal-venous phase, and a delayed-contrast phase). A Brilliance CT 64-multislice scanner (Philips Medical Systems, Best, The Netherlands) was used for scanning. An antecubital vein was injected with 120 mL of a contrast agent using a mechanical injector at a rate of 4.0 mL/s. The performed acquisition was performed using the following parameters: (1) rotation time = 0.75 s; (2) pitch = 0.984; and (3) slice thickness = 2.5 mm. The left portion in Figure 2 shows random samples from the dataset. There were three stages for each category (i.e., “non-responsive” and “responsive”). The three stages were pre-contrast, portal-venous, and delayed-contrast.

### 2.2. Methods

A manual delineation process was performed on the Wilms’ tumors images (from the three phases) by radiologists using in-house software, resulting in the construction of Wilms’ tumors’ ROIs. Samples from the dataset and their corresponding segmentations are presented in Figure 2.

**Feature extraction**

The outcomes of the machine learning model heavily rely on the extracted features [15,16]. This study used three groups of features: shape, functionality, and textural features. Shape features describe the morphological complexity of Wilms’ tumors based on spherical harmonics, descriptive elongation, and sphericity. Functionality-based features quantify the enhancement characteristics among the three contrast phases. Lastly, textural features capture the variant texture patterns within Wilms’ tumor through 1st-order histogram features and rotation invariant 2nd-order features based on (1) gray-level co-occurrence matrix (GLCM) and (2) gray-level run-length matrix (GLRLM). These features’ groups are described in the following section.

**Shape features** are used to describe the complexity of the identified WT within the kidney to enhance the sensitivity and specificity results of the response prediction [17,18]. As tumors with high growth rates and varying complex shapes have a high probability of being non-responsive to preoperative chemotherapy, usage of these features illustrates the complexity of the tumors, and accordingly, the response tendency. Utilization of shape descriptions will improve the capabilities of the automated predictions. However, it is critical to achieve accurate modeling for such an enhancement. For this project, extracting morphological features was crucial to diagnose Wilms’ tumors using descriptive shape features (namely, sphericity and elongation) and parametric state-of-the-art spectral analyses, employing SHs [19,20,21].

To start with the **spherical harmonics** approach, select an internal point in the Wilms’ tumor to be the spherical coordinate system origin. The tumor’s surface serves as a polar and azimuthal angle function represented by a linear combination of the $H_{\tau \beta }$ basis functions that are defined on the unit sphere. A triangulated mesh is constructed by spherical harmonics (SHs) to approximate the surface of the tumor, then apply unit sphere mapping. It provides accurate modeling using the attraction–repulsion method, as it holds the origin unit distance and every re-mapped node while retaining the distances between neighboring nodes [22]. Let the coordinates be $S_{\alpha ,n}$, with $∥S_{\left(\alpha ,n\right)}∥=1$, with an *n* node, and the iteration of the attraction–repulsion algorithm be α, where *n*∈1,…,N. In addition, let the displacement from node *n* to node *m* be denoted as $d_{\alpha ,mn}=S_{\alpha ,m}-S_{\alpha ,n}$, so that the Euclidean distance between the two nodes is ${\bar{d}}_{\alpha ,mn}=∥d_{\alpha ,mn}∥$. Finally, let $M_{n}$ be the set of index neighbors of node *N* in the triangulated mesh. Hence, the position for every node is updated through the attraction step to preserve it, centered with the corresponding neighbors, as shown in Equation (1) where $S_{a,1}$ and $S_{a,2}$ are attraction factor parameters of the algorithm.

(1) $$\begin{array}{c} S_{\alpha +1,n}^{{}^{\prime }}=S_{\alpha ,n}+S_{a,1}\sum_{m\epsilon M_{n}}\left(d_{\alpha ,mn}\times {\bar{d}}_{\alpha ,mn}^{2}+S_{a,2}\times \frac{d_{\alpha ,mn}}{{\bar{d}}_{\alpha ,mn}}\right) \end{array}$$

Subsequently, the repulsion step inflates all the mesh to prevent its degeneration, similarly to the attraction step, which allows the nodes to be closer to each other. This is shown in Equation (2), where $S_{r}$ is the repulsion factor parameter of the algorithm.

(2) $$\begin{array}{c} S_{\alpha +1,n}^{{}^{\prime \prime }}=S_{\alpha +1,n}^{{}^{\prime }}+\frac{S_{r}}{2N}\sum_{m=1;m\neq n}^{N}\frac{1}{{\bar{d}}_{\alpha ,mn}} \end{array}$$

Then, the unit sphere back projection is applied to the points, as shown in Equation (3).

(3) $$S_{\alpha +1,n}=\frac{S_{\alpha +1,n}^{{}^{\prime \prime }}}{\left|\right|S_{\alpha +1,n}^{{}^{\prime \prime }}\left|\right|}$$

During the attraction–repulsion algorithm terminal iteration $\alpha_{t}$, the kidney nodule surface is considered in one-to-one correspondence with the unit sphere. Each node of the original mesh $S_{n}$ = ($x_{n}$,$y_{n}$,$z_{n}$) is mapped with the corresponding point $S_{\alpha_{t},n}=(\sin\left(\theta_{n}\right)\times \cos\left(\varphi n\right),\sin\left(\theta_{n}\right)\times \sin\left(\varphi n\right),\cos\left(\theta_{n}\right)$, with a polar angle $\theta_{n}\in \left[0,\pi \right]$ and azimuthal angle φn∈[0,2×π). Later, there is a chance to explain the nodule using spherical harmonics series. With this performance, the lower-order harmonics give the nodule a rough depiction, whereas the higher-order harmonics give the surface finer details. Solving the isotropic heat equation for the surface of the nodule generates the SHs that are a function of the unit sphere. The $H_{\tau \beta }$ spherical harmonics with a degree of τ and an order of β is shown in Equation (4), where the SH factor is $s_{\tau \beta }$ and the associated Legendre polynomial is $L_{\tau }^{\left|\beta \right|}$, with a degree τ and an order of β.

(4) $$\begin{array}{c} H_{\tau \beta }=\left\{\begin{array}{cc} s_{\tau \beta }L_{\tau }^{\left|\beta \right|}\times \sin\left(|\beta |\varphi \right)\times \cos\left(\theta \right) & -\tau \leq \beta \leq -1\\ \frac{s_{\tau \beta }}{\sqrt{2}}L_{\tau }^{\left|\beta \right|}\times \cos\left(\theta \right) & \beta =0\\ s_{\tau \beta }L_{\tau }^{\left|\beta \right|}\times \cos\left(|\beta |\varphi \right)\times \cos\left(\theta \right) & 1\leq \beta \leq \tau \end{array}\right. \end{array}$$

**Figure 3:**
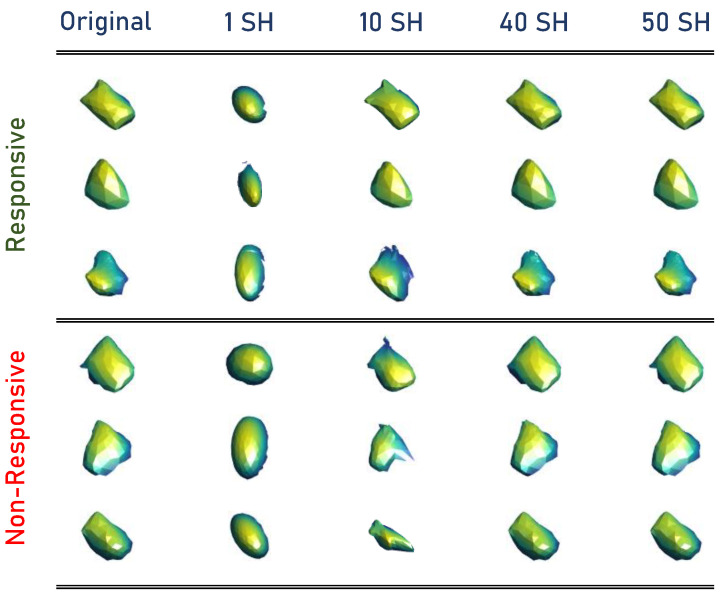
Samples from the constructed SHs.

Finally, using Equation (4), the Wilms’ tumor object is approximated through SHs. The higher-order combination SHs represent the tumors’ complexity. Thus, the total number of markers that quantify the identified tumor’s morphological complexity is the number of SHs used to approximate the original tumor, which was 50 for this work to modify the reconstruction of the tumors. After choosing a sufficient number, calculate the reconstruction error for each approximation between the approximated shape and original mesh. The original mesh of Wilms’ tumor is inherently aligned with the approximated shape mesh resulting from the unit sphere mapping for each approximation. Then, the sum of Euclidean distances between the corresponding nodes results in the total error between the two mesh models. By calculating the 50 approximations for each tumor, obtaining the reconstruction errors is essential to describe the morphology of Wilms’ tumors. Samples from the constructed SHs are presented in Figure 3.

(1) **Initialization Process:**
  − Triangulate the nodule surface.
  − Apply the Laplacian filtering to smooth the triangulated mesh.
  − The spherical parameterization is initialized using an arbitrary topology-preserving map onto the unit sphere.
  − Fix the $S_{a,1}$, $S_{a,2}$, $S_{r}$, and threshold *T* values.
(2) **Attraction-repulsion Process:**
  − **For every** α=0,1,…

    ∗ **For** n=1,…,N

      · Determine $S_{\alpha +1,n}^{\prime }$ using Equation (1)
    ∗ **For every** n=1,…,N

      · Determine $S_{\alpha +1,n}^{\prime \prime }$ using Equation (2)
      · Let $S_{\alpha +1,n}=S_{\alpha +1,n}^{\prime \prime }/∥S_{\alpha +1,n}^{\prime \prime }∥$
    ∗ **If**$\max_{n}∥S_{\alpha +1,n}-S_{\alpha ,n}∥\leq T$, **Then** let $\alpha_{t}=\alpha +1$ and **Terminate**.

**Sphericity** is a measure of the tumor region’s roundness in relation to a sphere. It is an arbitrary measurement that is not affected by scale or direction. This measure ranges from 0 to 1 with a 0-value indicating the roughest shape and a 1-value referring to a smooth sphere. It is worth mentioning that compared with other solids, a sphere has the least surface area for a given volume. It is calculated using Equation (5), where *A* and *V* denote the surface area and volume of the tumor.

(5) $$\mathrm{sphericity}=\frac{\sqrt[{3}]{36\times \pi \times V^{2}}}{A}$$

The ROI shape’s **elongation** reveals the link between the two biggest major components. It is calculated using Equation (6), where $\lambda_{1}$ and $\lambda_{2}$ refer to the second-largest and the largest axes, respectively. The values fall into a range of 0 to 1, with a 0-value indicating that the object is maximally elongated and a 1-value indicating that the cross-section between the first and second biggest primary moments is circular.

(6) $$\mathrm{elongation}=\sqrt{\frac{\lambda_{1}}{\lambda_{2}}}$$

**Functionality-based features**: the purpose of using intensity enhancement is to quantify Wilms’ tumors’ functionality. They are computed using changes in gray-level intensity across the contrast phases to extract 3 features to the enhancement changes between the 3 phases. The slopes are known as the change in gray-level intensity rate time for each phase. The non-responsive tumors showed more rapid slopes (i.e., higher absolute values) than those of the responsive ones.

**Textural features** are used to improve the specificity and sensitivity results of the prediction of Wilms’ tumor chemotherapy response. Textural analysis was applied using 1st- and 2nd-order textural features to express the heterogeneity/homogeneity of the extracted tumors from 3 different phases: delayed-contrast, pre-contrast, and portal-venous phase. The purpose of using these features was motivated from the knowledge that tumors with a heterogenous shape have a high probability of being non-responsive to preoperative chemotherapy. A normalized histogram shows the approximation of all the first-order texture characteristics. Second-order textural features were also used (i.e., GLCM and GLRLM) due to first-order texture being sensitive to noise [19,23,24]. The main reason for using both types was to detect the inhomogeneity in WTs.

**GLCM** clarifies how frequently the values of a pair of gray-level intensities appear adjacently within the object. It is a matrix that takes into account the spatial relationships between a neighborhood block’s reference and surrounding voxels. It determines the frequencies of each pair of gray levels based on the range of the target object’s gray levels. Construction for the GLCM begins by specifying the gray-level range of Wilms’ tumor objects and normalizing the observed values of the gray level to the aimed range. Later, every possibility of each pair is determined and illustrated in rows and columns of the matrix (every element in the matrix is related to two values of gray levels representing the element row and column). Lastly, we compute the value of each element by examining the difference between each voxel and its neighbors. The neighborhood block is determined by a distance $<\sqrt{2}$ that leads to the calculations of the rotation invariant. During the analysis phase, the gray-level values were normalized to a range of [0,255], resulting in a GLCM of 256×256. After constructing GLCM, we normalize a matrix by summing all elements = 1 to extract the distinct textural features. The extracted six features were correlation, energy, second angular moment, dissimilarity, contrast, and homogeneity.

**Figure 4:**
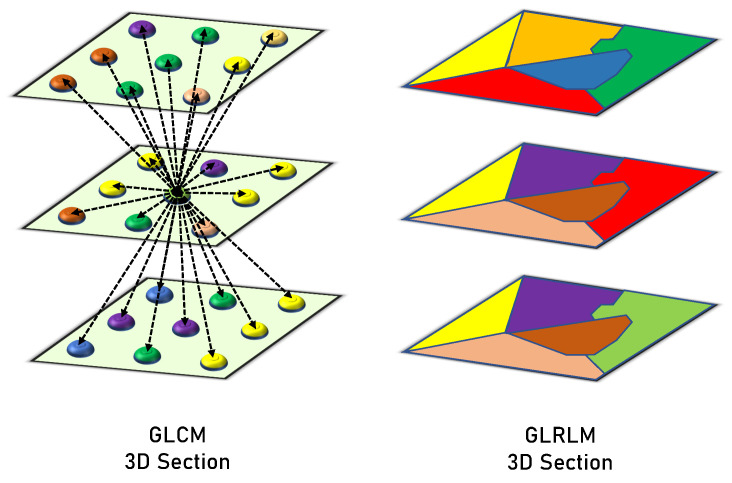
Graphical comparison between the GLCM and GLRLM.

**GLRLM** examines the voxel runs to measure the voxel connectivity. It counts how many times a row of voxels containing a certain gray-level value has occurred. The gray-level range is represented by the number of rows in this matrix, whereas the highest run and greatest object dimension are represented by the number of columns. As a result, the frequency of a certain gray-level value is shown by each member in the matrix for a given consecutive voxel run length. Every structure has a matrix of 256 rows (gray-level normalized range), and the number of columns varies among the objects. In this case, the main concern is the runs that have consecutive horizontal voxels in the XY-plane (of the same layer), and the vertical voxel runs are examined in the Z-axis (among several layers). Finally, the different computed measures of GLRLM are used to describe the textures of the structures. The following 16 features that are extracted from the GLRLM quantify the gray level run: Long and Short Run Emphasis (shortly, LRE and SRE), Gray Level Non-Uniformity (GLN), Run Length Non-Uniformity (RLN), Gray-Level Variance (GLV), Run Percentage (RP), Run Entropy and Variance (RE and RV), High and Low Gray Level Run Emphasis (HGLRE and LGLRE), Short Run High and Low Gray Level Emphasis (SRHGLE and SRLGLE), and Long Run High and Low Gray Level Emphasis (LRHGLE and LRLGLE). Figure 4 presents a graphical comparison between the GLCM and GLRLM.

**Classification and Optimization**

The extracted features characterizing the tumors are integrated using fusion to predict their response to preoperative chemotherapy. These integrated sets of features are then fed to an ML-based SVM classification model. Each algorithm has its own hyperparameters, and hence, grid search (GS) is used to compute the optimum values of the different hyperparameters. The optimal hyperparameters of the classification model were found to be the quadratic kernel, tolerance = 0.001, maximum iterations = unlimited, γ=0.001, degree = 3, decision function shape “ovr” (i.e., one-vs-rest), cache size = 200, no tie breaking, and regularization parameter = 1 [25].

**Figure 5:**
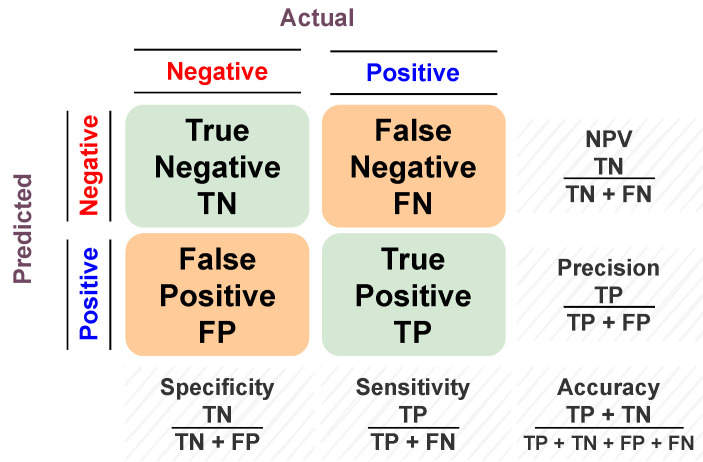
Graphical summarization of the confusion matrix.

To assess the model’s predictive ability, different performance metrics are estimated. Accuracy, sensitivity, specificity, and f1-scores are examples of these performance metrics. They all belong to so-called overlap-based metrics. They are based on the basic cardinalities of the confusion matrix (i.e., TP, FP, FN, and TN) between the predicted and actual value. Accuracy is the proportion of correct predictions to the total input samples. The sensitivity (i.e., recall or true positive rate (TPR)) is the metric that measures the model’s capability to produce true positive predictions of each available category. In other words, it is the ratio of positive points that are correctly classified as positive out of all positive points. Similarly, specificity (i.e., true negative rate (TNR)) assesses the model’s capability to produce true negative predictions of each available category. In other words, it is the ratio of negative points that are correctly classified as negative out of all negative points. The F1 score (i.e., dice coefficient or overlap index) is the harmonic mean between precision and recall, and it ranges from 0 to 1. It indicates how many instances were correctly classified (i.e., how precise the classifier is), along with ensuring that it does not ignore a large portion of samples. A graphical summarization of the confusion matrix is presented in Figure 5.

Cross-validation is referred to as out-of-sample testing. Its overall aim is to assess ML algorithms by training them on different subsets derived from the input dataset. Additionally, it can be applied to detect overfitting, which implies that the model is not generalizing patterns effectively in unseen data. K-fold cross-validation refers to a case when the dataset is split into a *K* number of folds. *K* indicates the number of sets the original dataset is split into. Thus, each fold is utilized as a testing set at one point in the process. Using K-fold cross-validation allows all data parts to be represented in both training and testing data, leading to better evaluations of the performance of our model. Additionally, using k-fold cross-validation, more models will be producing more results. For instance, if the k-value is set to 4, 4 different models will be trained, and hence, 4 different results will be available to be used for evaluation of the performance of the model. In this study, we used 3 different cross-validation approaches, namely, leave-one-subject-out (LOSO), 4-fold, and 10-fold.

## 3. Experiments and Results

To highlight the added value of this system, as discussed earlier, the current study suggests a CAP system that predicts the response of childhood solid renal tumors to preoperative chemotherapy based on tumor imaging characteristics extracted from preoperative contrast-enhanced CT scans, as shown in Figure 1. The CAP utilizes the CT images across three phases. It extracts first- and second-order textural features, as well as shape and functionality-based features, as shown in Table 1.

Using a tuned SVM classifier, the performance of the suggested CAP system was evaluated utilizing these extracted features. To highlight the effectiveness of integrating the individual markers, the performance of these individual models was compared with the diagnostic abilities of a model utilizing the integrated group of features. As shown in Table 2, utilizing the integrated features with the CAP system outperformed using individual groups of features in terms of all evaluation metrics. This featural integration fused different characteristics of the tumors to get a defining value of each group, which led to improved diagnostic performance. For the individual groups of features, the second-order textural features achieved the highest accuracy (92.06%) among all groups. The GLCM had the highest sensitivity (95.65%), and the spherical harmonics outperformed other groups in terms of specificity (94.14%). The highest F1-score was 0.95, which was reported by the GLCM and GLRLM groups. The texture feature groups reported better results than the other groups. By integrating all features, the accuracy improved to 95.24% and the F1-score improved to 0.97. The sensitivity and specificity remained 95.65% and 94.12%, respectively.

To highlight the robustness and reproducibility of the suggested approach, the experiments were performed using stratified 4-fold, 10-fold, and LOSO cross-validation approaches. For each validation, different ML classifiers, namely, *K*-Nearest Neighbor (KNN), Decision Tree (DT), Random Forest (RF), Logistic Regression (LR), Multi-Layer Perceptron (MLP), and SVM, were trained on the integrated group of extracted features and tuned using the GS to select the optimal hyperparameters. It is worth mentioning that for both 10-fold and 4-fold approaches, the experiments were repeated 10 times, and the evaluation metrics were reported in terms of means and standard deviations. For the LOSO (reported in Table 3), with an accuracy, sensitivity, specificity, and F1-score of 95.24%, 95.65%, 94.12%, and 0.97, respectively, the SVM outperformed all other ML classifiers. It achieved at least 5% higher specificity than other models.All accuracy, F1-score, and sensitivity results were above 90%. The results of the 10-fold approach are tabulated in Table 4. All the utilized classifiers achieved inferior results compared with those obtained by the SVM. Aside from the SVM, the RF classifier outperformed other classifiers in both accuracy and sensitivity, but was limited by its relatively low specificity. The SVM and LR models reported the lowest standard deviations, which were 0%. The highest accuracy was 93.65% by the SVM model, the highest sensitivity was 9565% by the SVM model, the highest specificity was 88.24% by the SVM, LR, and KNN models, and the highest F1-score was 0.96 by the SVM model.

For the 4-fold approach (Table 5), the SVM also achieved the highest diagnostic performance. Both KNN and LR showed high specificity, but their accuracy and sensitivity were almost the lowest among the incorporated classifiers.

## 4. Discussion

Following discussions with the medical team, we were motivated by the fact that non-responsive tumors to preoperative chemotherapy showed a heterogenous appearance, complex shapes, and rapid enhancement across the three contrast phases. Hence, we decided on characterizing Wilms’ tumors before chemotherapy by extracting shape, texture, and functionality-based features. Textural features were used to evaluate the inhomogeneity of tumor lesions. Textural analysis incorporated mathematical equations to evaluate the gray-level spatial variation in images to provide a quantitative analysis of the tumor’s texture. Textural analysis was incorporated within our study in the form of first- and second-order (i.e., GLCM and GLRLM) features. Moreover, parametric spectral analysis employing spherical harmonics was used, along with other shape features, to capture multiple aspects of the histological formation of the tumors. To validate performance, different cross-validation approaches were used in the experiments. Multiple tuned ML-based classification models were utilized on the retrieved groups of features. Firstly, each individual group of features was used, along with the classification models, to reach the final decision. The features were then integrated together, and the final prediction was made utilizing these integrated features. Various performance measures were incorporated to report on the overall performance. The SVM classifier, along with the integrated group of features, outperformed other ML models.

## 5. Limitations

The current study limitations included: (1) the limited size of the dataset; (2) the focus on only machine learning algorithms; and (3) classifying the cases into only two categories. We suggest that these limitations be addressed in future work.

## 6. Conclusions and Future Work

The developed CAP system integrated texture, shape, and functionality-based features, which led to an impressive predictive performance (accuracy = 95.24%) using an SVM classification model. The obtained experimental results demonstrate the viability of integrating different significant features representing diverse characteristics of Wilms’ tumors to make early predictions on tumor response to preoperative chemotherapy.

In future work, we aim to use a larger dataset. The classification of the preoperative response into more categories (i.e, static, regressive, and progressive) is another area to be addressed. Moreover, deep learning methods could be utilized with the help of convolution neural networks (CNNs) and data augmentation techniques to perform automatic segmentation of Wilms’ tumors [26,27].

## Figures and Tables

**Table 1:** The number of entries for each group of extracted features and the phase they are extracted from.

Features	Contrast-Phase	Number of Features
**Texture Features**		
Histogram-based (first-order)	Venous	26
GLCM (second-order)	Pre + venous + delayed	18 (6 per phase)
GLRLM (second-order)	Pre + venous + delayed	36 (12 per phase)
**Shape Features**		
Spherical harmonics	Venous	50
Descriptive	Venous	2
**Functionality-Based Features**		
Enhancement slopes	Pre + venous + delayed	2
**All**		
Integrated	Pre + venous + delayed	134

**Table 2:** Comparison of the suggested CAP system’s diagnostic performance using the individual features with a tuned SVM classifier, looking at accuracy, specificity, sensitivity, and F1-scores.

Features	Accuracy %	Sensitivity %	Specificity %	F1-Score
**Texture Features**				
Histogram (first-order)	87.30	89.13	82.35	0.91
GLCM (second-order)	92.06	95.65	82.35	0.95
GLRLM (second-order)	92.06	93.48	88.24	0.95
**Shape Features**				
Spherical harmonics	90.84	89.13	94.12	0.93
Descriptive	90.84	91.30	88.24	0.93
**Functionality-Based Features**				
Enhancement slopes	88.89	89.13	88.24	0.92
**All**				
Integrated	95.24	95.65	94.12	0.97

**Table 3:** The evaluation metrics of different ML classifiers using LOSO cross-validation approach, along with the integrated group of extracted features.

Classifier	Accuracy %	Sensitivity %	Specificity %	F1-Score
**DT**	90.48	93.48	82.35	0.93
**KNN**	92.06	93.48	88.24	0.95
**LR**	90.48	91.30	88.24	0.93
**MLP**	92.06	93.48	88.24	0.95
**RF**	92.06	95.65	82.35	0.95
**SVM**	95.24	95.65	94.12	0.97

**Table 4:** The evaluation metrics (reported in mean ± standard deviation after 10 repetitions) of different ML classifiers using stratified 10-fold cross-validation approach, along with the integrated group of extracted features.

Classifier	Accuracy %	Sensitivity %	Specificity %	F1-Score
**DT**	89.95 ± 1.98	92.03 ± 3.69	84.31 ± 2.77	0.93 ± 0.02
**KNN**	89.42 ± 0.75	89.86 ± 2.71	88.24 ± 4.80	0.93 ± 0.01
**LR**	88.89 ± 0.00	89.13 ± 0.00	88.24 ± 0.00	0.92 ± 0.00
**MLP**	89.95 ± 0.75	92.03 ± 3.69	84.31 ± 7.34	0.93 ± 0.01
**RF**	91.01 ± 0.75	94.20 ± 2.71	82.35 ± 4.80	0.94 ± 0.01
**SVM**	93.65 ± 0.00	95.65 ± 0.00	88.24 ± 0.00	0.96 ± 0.00

**Table 5:** The evaluation metrics (reported in mean ± standard deviation after 10 repetitions) of different ML classifiers using stratified 4-fold cross-validation approach, along with the integrated group of extracted features.

Classifier	Accuracy %	Sensitivity %	Specificity %	F1-Score
**DT**	85.71 ± 2.24	89.13 ± 3.07	76.47 ± 8.32	0.90 ± 0.02
**KNN**	86.77 ± 2.70	86.96 ± 4.70	86.27 ± 7.34	0.91 ± 0.02
**LR**	84.66 ± 2.70	85.51 ± 2.71	82.35 ± 4.80	0.89 ± 0.02
**MLP**	85.71 ± 1.30	89.13 ± 3.07	76.47 ± 9.61	0.90 ± 0.01
**RF**	88.36 ± 1.50	91.30 ± 3.07	80.39 ± 7.34	0.92 ± 0.01
**SVM**	91.01 ± 1.98	91.30 ± 1.77	90.20 ± 2.77	0.94 ± 0.01

## Data Availability

Data can be made available after acceptance upon reasonable request to the corresponding author.
